# [^68^Ga] Ga-Pentixafor diffuse bilateral Adrenal & Breast uptake in a patient with High-grade Glioma: A note of caution on normal variants

**DOI:** 10.22038/AOJNMB.2022.66223.1458

**Published:** 2023

**Authors:** Hessamoddin Roustaei, Emran Askari, Somayeh Barashki, Kazem Anvari, Ramin Sadeghi, Kamran Aryana

**Affiliations:** 1 Nuclear Medicine Research Center, Mashhad University of Medical Sciences, Mashhad, Iran; 2 Cancer Research Center, Faculty of Medicine, Mashhad University of Medical Sciences, Mashhad, Iran

**Keywords:** CXCR4, High-grade Glioma, Adrenal, Breast, PET/CT

## Abstract

[^68^Ga] Ga-labeled C-X-C motif receptor4 as a novel radio-ligand using PET/CT has been investigated for tracing various kinds of solid and hematopoietic malignancies in recent years. High-grade Glioma (WHO classification 2016 grade III and IV) shows elevated levels of CXCR4 ligand expression in the affected tumoral cells. Healthy and non-affected organ cells express low-level CXCR4 ligands density. We performed [^68^Ga] Ga-Pentixafor (Pars-Cixafor™) PET/CT in a patient with high-grade Glioma (anaplastic oligodendroglioma WHO grade III) with no other documented medical condition and history. In addition to the Pentixafor-avid tumor remnant in the PET/CT images, we observed mild symmetrical bilateral uptake in the fibro glandular tissue of the breasts and moderate CXCR4(Pentixafor) avidity in both adrenal glands without any discernable pathology and abnormal density changes in the CT component of the study. Attention should be paid to the interpreting [^68^Ga] Ga-Pentixafor PET/CT examination and its normal uptakes and variants.

## Introduction

 [^68^Ga] Ga-labeled C-X-C motif receptor 4 as a novel radio-ligand using PET/CT has been investigated for tracing various kinds of solid and hematopoietic malignancies e.g., High-grade Glioma that shows elevated levels of CXCR4 expression. Information regarding potential pitfalls and various normal variants once interpreting [^68^Ga] Ga-Pentixafor PET/CT are limited.

***Case Report***

 A Thirty-three-year-old woman with a history of treated anaplastic oligodendroglioma (WHO III) in the left occipito-parietal mass with spread to the lateral ventricle and basal ganglia was referred due to worsening headaches and new-onset seizure. She was referred to our department for [^68^Ga] Ga-CXCR4 PET/CT. 

 Imaging was performed on a 6-slice dedicated PET/CT scanner 60 minutes post-injection of 4.03 mCi (149.11 MBq) of radio-labeled ^68^Ga-CXCR4. Radio-labeling was done under GMP regulations via a fully-automated labeling module with ^68^Ga eluted from PARS-GalluGEN ^68^Ge/^68^Ga generator. Diagnostic CT acquisition (130 Kv, 240mAs, slice thickness 3 mm, 512×512 matrix size, increment of 1.5 mm/s, rotation time of 1.0s, and pitch index of 0.55) was made with no contrast, followed by single bed PET imaging. 

## Discussion

**Figure 1:**
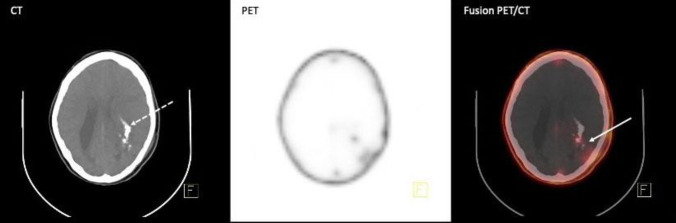
Brain [^68^Ga] Ga-Pentixafor PET/CT. mild CXCR4 avidity (SUV_max_: 1.68) in the left occipito-parietal lobe (**solid arrow**) with evidence of adjacent calcification (**dotted arrow**)

 The scan revealed mild CXCR4 avidity (SUV_max_: 1.68) in the left occipito-parietal lobe (solid arrow) with evidence of adjacent calcification (dotted arrow) and good tumor-to-background ratio (Figure 1), as we expected in grade III, compatible with the tumoral remnant. No abnormal activity in the rest of the brain was noticed. 

**Figure 2:**
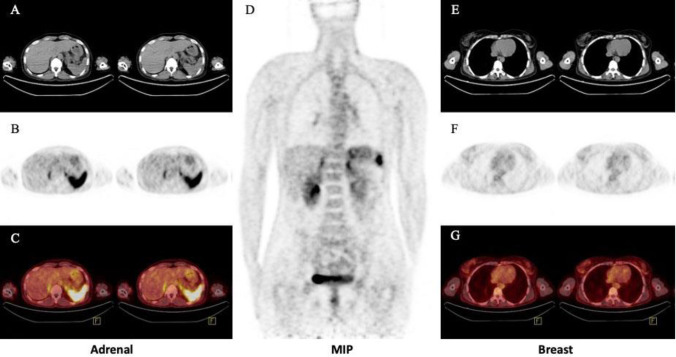
[^68^Ga] Ga-Pentixafor PET/CT. depicting slices of adrenals and breasts normal uptake

 The acquisition was continued in skull-base to mid-thigh regarding study design with low-dose CT for attenuation correction and anatomical correlation. The whole-body PET/ CT images showed mild diffused-bilateral breast uptake (SUV_max_: 1.92) corresponding to fibro-glandular tissue on CT slices (Figure 2, E, F, G). Moreover, both adrenal glands revealed uniformly elevated CXCR4 avidity (SUV_max_: 5.82) with no corresponding CT abnormality (Figure 2 A-D).

 [^68^Ga] Ga-labeled CXCR4-directed PET/CT imaging in high-grade Glioma has shown promising information regarding residual remnant and aggressiveness of tumor (1, 2).

 [^68^Ga] Ga-labeled C-X-C motif receptor4 as a novel radio-ligand using PET/CT has been investigated for tracing various kinds of solid and hematopoietic malignancies (3, 4). 

 Although breast cancer has shown over-expression of CXCR4; normal healthy breast tissue expressed low-density CXCR4 receptors (5- 7). 

 Low level over-expression of CXCR4 ligands in the fibroglandular tissue of our case can be due to hormonal stimulation and during the menstrual cycle (our patient was in the follicular phase; day 3).

 In contrast to normal adrenal tissue, significantly elevated levels of CXCR4 have been reported in cortisol-producing and aldosterone-producing adenomas, as well as adrenal hyperplasia (8-10). Nevertheless, our patient had no prior history of hypertension, neither relevant family nor drug history. The blood lab data (Potassium, Sodium levels and Bicarbonate) were within normal limits as well. To sum up, tracer uptake in both breasts and adrenals can be considered normal variants. Thus, attention should be paid to these normal uptakes while interpreting [^68^Ga] Ga-CXCR4 PET/CT examination.

## Compliance with ethical standards

 The patient was enrolled in a clinical trial of CXCR4-targeted PET/CT in vivo assessment of high-grade gliomas (Ir. MUMS. Medical. REC. 1399.734). All procedures performed in studies involving human participants were in accordance with the ethical standards of the institutional research committee and with the 1964 Helsinki declaration and its later amendments or comparable ethical standards.

## Conflict of interest

 The authors declare that they have no conflict of interest.

## Informed consent

 Written Informed consent was obtained from our patient.

## Funding

 This research did not receive any specific grant from funding agencies in the public, commercial, or not-for-profit sectors. 

## References

[1] Lapa C, Lückerath K, Kleinlein I, Monoranu CM, Linsenmann T, Kessler AF (2016). 68Ga-pentixafor-PET/CT for imaging of chemokine receptor4 expression in glioblastoma. Theranostics..

[2] Jacobs SM, Wesseling P, de Keizer B, Tolboom N, Ververs FF, Krijger GC (2022). CXCR4 expression in glioblastoma tissue and the potential for PET imaging and treatment with [68Ga] Ga-Pentixafor/ [177Lu]Lu-Pentixather. European journal of nuclear medicine and molecular imaging..

[3] Kircher M, Herhaus P, Schottelius M, Buck AK, Werner RA, Wester HJ (2018). CXCR4-directed theranostics in oncology and inflammation. Annals of Nuclear Medicine..

[4] Herrmann K, Lapa C, Wester HJ (2015). Biodistribution and radiation dosimetry for the chemokine receptor CXCR4-targeting probe 68Ga-pentixafor. J Nucl Med..

[5] Vag T, Steiger K, Rossmann A, Keller U, Noske A, Herhaus P (2018). PET imaging of chemokine receptor CXCR4 in patients with primary and recurrent breast carcinoma. EJNMMI research..

[6] Zhang Z, Ni C, Chen W, Wu P, Wang Z, Yin J (2014). Expression of CXCR4 and breast cancer prognosis: a systematic review and meta-analysis. BMC cancer..

[7] Laird SM, Widdowson R, El-Sheikhi M, Hall AJ, Li TC (2011). Expression of CXCL12 and CXCR4 in human endometrium; effects of CXCL12 on MMP production by human endometrial cells. Human reproduction..

[8] Ding J, Zhang Y, Wen J, Zhang H, Wang H, Luo Y (2020). Imaging CXCR4 expression in patients with suspected primary hyper-aldosteronism. European journal of nuclear medicine and molecular imaging..

[9] Heinze B, Fuss CT, Mulatero P, Beuschlein F, Reincke M, Mustafa M (2018). Targeting CXCR4 (CXC chemokine receptor type 4) for molecular imaging of aldosterone-producing adenoma. Hypertension..

[10] Ding J, Tong A, Zhang Y, Zhang H, Huo L (2021). Cortisol-producing adrenal adenomas with intense activity on 68Ga-pentixafor PET/CT. Clinical Nuclear Medicine..

